# The Abuse of Quinine

**Published:** 1890-09

**Authors:** W. C. Bilbro

**Affiliations:** Murfreesboro, Tenn.


### THE ABUSE OF QUININE.*

By W. C. BILBRO, M. D., MURFREESBORO, TENN.

But little has been written concerning the toxic effect of quinine, and yet cases are not uncommon in which we may trace disease to the abuse of this popular drug.

The laity load themselves down with the drug without any reason other than the idea that they must take something and that the medical profession has educated them to do it, by its habit of mechanically saying to every one who complains—*take a few doses of quinine.*

If all the rattled nervous systems and demoralized stomachs, coupled with deafness and other maladies, were to testify truthfully, they would unfold a tale that would harrow up the soul.

No one of any experience will deny the toxic effect of quinine in certain conditions, and, too, conditions that it is often prescribed in.

*Read before the Tennessee State Medical Society, in Memphis, April, 1890.

But in order to more fully understand the ill effects, we will determine first what are its physiological ones. Its most important physiological effect is its anti-periodic effect, so beautifully shown in remittent and intermittent fevers. It also has a remarkable power to arrest the migratory movements of leucocytes, and for this reason is often prescribed in pneumonia ; but let us see if another condition does not arise that will be likely to cause the administration of it to do more harm than good. Our patient's breathing capacity is diminished already by reason of the fact that the air-cells of the inflamed portion are impervious, therefore making it very difficult for him to get enough oxygen; then we naturally desire to steer clear of every remedy that is in the least calculated to increase discomfort and danger.

What do we do by administering quinine ? Bartholow says that the salts of cinchonia diminishes the power of the red corpuscles to absorb oxygen and decreases the amount of carbonic acid given off. Then again, the activity of the respiratory center is diminished ; and no one will deny the possibility of determining the action of drugs on this center as a basis on which to found our treatment. I am sure that in more than one case, I have observed very untoward symptoms from a large dose of quinine in inflammation of the lungs. I have in mind now a lady who was almost asphyxiated from taking a ten-grain dose of quinine while she had a pneumonitis. I am sure that had she repeated it, with the combined sedative effect on the respiratory center and the diminished oxygenating power, she would have died. Brown-Sequard says that very large amounts will sometimes completely paralyze the pneumogastric nerve.

In the spring of 1885 I was hurriedly called to see a little child of ten years of age, who had been having an intermittent fever, and of course quinine was administered, *ad libitum.* I arrived in time to get this history, for the child was dead. About two hours previous to the time the mother sent for me she gave the child a dose of quinine. She did not weigh the amount she gave the child, but guessed it to have been about ten grains. The little fellow was out running about the yard seemingly well, when suddenly he came into the house and said he felt badly and laid down, and shortly after died.

Another case I have in mind in which a complete suspension of reflex sensibility, with incontinence of urine, was brought about by the continued use of quinine, which was quickly relieved by simply discontinuing its use.

I believe I have a case of paraplegia under my care, now that was brought about by the quinine habit. My patient had been in the habit of taking a bottle or more of cinchonidia a week for some time, when he observed that the power of locomotion was being diminished, which gradually increased until complete paraplegia resulted.

I do not think it should be given in any case where there is any likelihood of the stomach becoming irritated, and for this reason I have abandoned it in dysentery per orem. I have, however, given it by injection with the best results.

I had a case of acute mania the direct result, I believe, of large and continued doses of quinine, the patient having taken the drug for several days for a cold. He became so nervous that it caused insomnia for several days, which brought about the condition. In this case it could have been nothing else but the quinine, for he never had any fever from first to last of his sickness. The result of a discontinuance was complete recovery in three or four days, with only a hypodermic administration of one-half grain dose of morphia for the time mentioned.

Then again, I believe the continued abuse of it will ultimately do away with its legitimate use, just as blood-letting has almost become obsolete from its abuse.

Let us examine our cases more carefully and recognize the fact that quinine is potent for evil as well as good ; that like every other drug in our medical armamentarium, it should be used with discretion and should not be trusted to the unskillful and used merely to have the appearance of doing something. It seems to me that we, as medical men, should condemn uncompromisingly the habit of prescribing any drug indiscriminately. It is a fact that nearly every family keeps and uses quinine continuously for any and every real and imaginary trouble, and it is the direct result of the careless way physicians prescribe it. The older members of the profession are well aware of the fact that just such abuse of blood-letting has been one of the most potent factors in its discontinuance, and we are thereby deprived of one of our most useful agencies. As an antipyretic, I consider it to be admissible only in a few conditions, mainly those in which there is a malarial agency to combat. There are other medicines which, under my observation, are much more reliable as febrifuges and are not fraught with such evil; others that will not so demoralize our patients' stomachs and nervous system.

In the recent epidemic of influenza it was advocated by some to be the remedy *par excellence*, and if I have ever noticed any good effects from it I do not remember it now. I find that it increases the nervous symptoms without any appreciable good arising from its administration.

